# Upregulation of IL-1 Receptor Antagonist in a Mouse Model of Migraine

**DOI:** 10.3390/brainsci9070172

**Published:** 2019-07-19

**Authors:** Salvo Danilo Lombardo, Emanuela Mazzon, Maria Sofia Basile, Eugenio Cavalli, Placido Bramanti, Riccardo Nania, Paolo Fagone, Ferdinando Nicoletti, Maria Cristina Petralia

**Affiliations:** 1 Department of Biomedical and Biotechnological Sciences, University of Catania, Via S. Sofia 89, 95123 Catania, Italy; 2 IRCCS Centro Neurolesi Bonino Pulejo, Strada Statale 113, C.da Casazza, 98124 Messina, Italy

**Keywords:** migraine, IL-1RN, cortical spreading depression, mouse model

## Abstract

Migraine is a disorder characterized by attacks of monolateral headaches, often accompanied by nausea, vomiting, and photophobia. Around 30% of patients also report aura symptoms. The cause of the aura is believed to be related to the cortical spreading depression (CSD), a wave of neuronal and glial depolarization originating in the occipital cortex, followed by temporary neuronal silencing. During a migraine attack, increased expression of inflammatory mediators, along with a decrease in the expression of anti-inflammatory genes, have been observed. The aim of this study was to evaluate the expression of inflammatory genes, in particular that of IL-1 receptor antagonist *(IL-1RN)*, following CSD in a mouse model of familial hemiplegic migraine type 1 (FHM-1). We show here that the expression of *IL-1RN* was upregulated after the CSD, suggesting a possible attempt to modulate the inflammatory response. This study allows researchers to better understand the development of the disease and aids in the search for new therapeutic strategies in migraine.

## 1. Introduction

Migraine is a common neurological disease, representing the fourth cause of years lived with disability (YLDs) for women, and the eighth for men [1,2,3]. It is characterized by attacks of monolateral headaches, associated with nausea as well as phono- and photophobia [4]. Around 30% of patients also report aura symptoms, which are mostly visual. The cause of the aura is believed to be related to the cortical spreading depression (CSD), a self-propagating wave of cellular depolarization from the occipital cortex, followed by a transitory neuronal silencing [5,6]. 

The manifestation of these attacks depends on a genetic predisposition, associated to environmental stimuli such as stress, hormones, meteorological changes, and sleep disorders. For this reason, it is difficult to identify the etiology and physiopathogenesis of migraine. Recent evidence also suggests that immunoinflammatory events may also play a role in the pathogenesis of migraine [7,8]. 

In particular, much attention has been given to the role of immune-system hormones, named cytokines, to the pathogenesis of migraine [9].

Based on preclinical and clinical studies, the cytokines have been divided into at least five subfamilies: the proinflammatory Th1/Th17 cytokines, the anti-inflammatory Th2/Th3 cytokines, and the Th9 cytokine, represented by IL-9 [10,11,12].

It has been shown that Th1 and Th17 cytokines primarily exert proinflammatory effects. They are produced by M1 macrophages, Th1 and Th17 cells, and include—among others—IL-1, TNF-alpha, IFN-gamma, IL-12, IL-18, IL-22, IL-23, and IL-17. They may contribute to the initiation of cell-mediated autoimmune diseases such as rheumatoid arthritis, type 1 diabetes, multiple sclerosis, and Guillain-Barré syndrome [10,11,13].

Anti-inflammatory cytokines (e.g., IL-4, IL-10, IL-13 IL-35, TGF-beta) are primarily produced from M2 macrophages and Th2 and Th3 cells. They decrease Th1- and Th17-mediated immunoinflammatory events, and are implicated in IgE-mediated allergic diseases and eosinophil-mediated pathologies [14,15,16,17]. 

Sometimes, such as in the case of systemic lupus erythematosus, it seems that the combined action of Th1/Th2 cytokines may be simultaneously involved in the pathogenesis of the disease [18]. The precise role of IL-9 in regulation of the immune responses is less well-defined, and is receiving a great deal of attention [12]. 

In addition, the function of cytokines is also finely regulated by endogenous antagonists, which have been described for most cytokines, such as soluble receptors, anti-cytokine autoantibody, and for IL-1, the IL-1 receptor antagonist [19,20,21]. These endogenous antagonists are usually produced during immune responses, and serve to control and downregulate excessive signaling of the cytokine through binding with its functionally active receptors expressed on the surface of the target cells. For example, we have shown that blood levels of IL-1ra are augmented during attacks of multiple sclerosis and are further augmented from the treatment of the patients with IFN-beta [22].

Cytokines have recently been associated to the etiology of migraine, even if conflicting results have been found [23,24,25]. The association between migraine and the interleukin-1 receptor antagonist variable number tandem repeat (IL-1RN VNTR) has previously been investigated, but no statistically significant differences were discovered [26].

A cross-talk between neurons and immune cells has also been reported, which may contribute to the generation of the pain [27]. Indeed, activated macrophages and other non-neuronal cells might induce a meningeal ‘‘sterile inflammation’’ [28,29], contributing to the pain symptoms [30,31]. Furthermore, it has been reported that inflammation in the trigeminal nerve territory is often observed during migraine attacks [32], so the acute administration of corticosteroids has been tested to block pain [33]. 

Familial hemiplegic migraine type 1 (FHM-1) is a monogenic type of migraine with aura caused by mutations in the *CACNA1A* gene which determine an alteration of the passage of Ca^2+^ ions in the cerebral cortex [34,35]. The FHM mouse model, generated by introducing the R192Q mutation into the endogenous *CACNA1A* gene [36], is used to study the physiopathology of FHM-1 [37,38]. R192Q KI mice have increased neuronal Ca^2+^ influx and augmented glutamate release [39], which may explain the increased susceptibility to CSD [36,39]. Data from animal models have shown that CSD is able to activate meningeal trigeminovascular neurons, generating the sensation of pain [40,41,42]. 

The aim of the present study was to determine the expression of inflammatory genes and in particular that of IL-1 receptor antagonist (*IL-1RN*) upon CSD, in a murine model of FHM1. 

## 2. Materials and Methods 

### 2.1. Dataset Selection and Analysis

In order to evaluate the brain expression levels of *IL-1RN* following CSD, we interrogated the GSE67933 dataset, obtained from the GEO dataset [43]. The dataset included whole-genome transcriptomic data from wild-type (WT) mice and transgenic mice carrying the CACNA1A R192Q missense mutation (FHM1 R192Q mice). Gene expression profiles were obtained from cortical tissue of FHM1 R192Q and control mice, 24 h after experimentally induced CSD [44]. Briefly, CSD was induced by seven applications of a cotton pellet soaked in 300 mM KCl on the dura overlaying the occipital cortex, while in sham animals 300 mM NaCl was applied instead of KCl [44]. Deep SAGE sequencing was used to generate the expression profiles, and the data were normalized using the trimmed mean of M-values (TMM) method.

Functionally correlated genes were obtained using the web-based software STRING [45] and visualized as a gene network. Relationships of genes in the network were defined in terms of co-expression, text-mining, biochemical data, curated pathway, and protein–protein interactions. The confidence cut-off for showing interaction links was set as medium (0.4) and the maximum number of interactors in the first shell was set at 50. 

Heatmapper [46] software was used to generate the expression heatmap of the functional related genes to *IL-1RN* and to perform hierarchical clustering of genes. Average linkage was used as clustering method and Euclidean distance as distance measurement. 

Co-expression analysis was carried out using the CoExpress software [47], and gene enrichment for biological processes (BPs) and molecular functions (MFs) was performed using the web-based utility DAVID version 6.8 [48].

### 2.2. Statistical Analysis

Data are shown as mean ± standard deviation. Differences in *IL-1RN* expression among the experimental groups were evaluated by ANOVA (Student’s *t*-test) with Bonferroni post-hoc test. Correlation analysis was performed using the Pearson’s correlation test. Statistical analysis was performed using IBM SPSS Statistics 23 [49] and GraphPad Prism 8.0.2 [50]. 

## 3. Results

### 3.1. IL-1RN Expression after CSD

**Figure 1:**
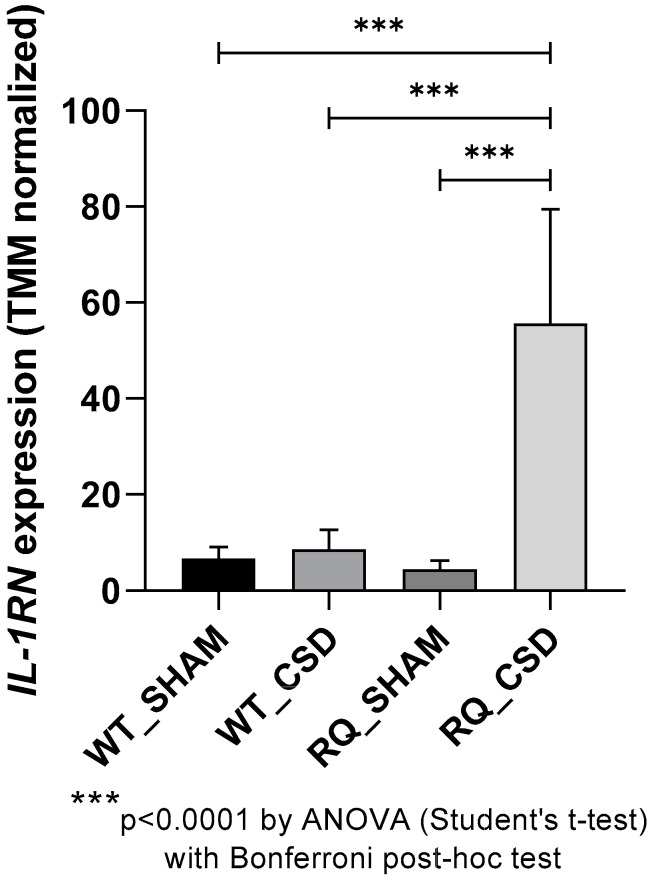
*IL-1RN* expression in a model of migraine. The expression of *IL-1RN* was investigated in mice bearing the R192Q mutation and wild–type (WT) mice at baseline and following CSD, as determined in the GSE67933 dataset. Data are presented as trimmed mean of M-values (TMM) normalized expression.

The GSE67933 dataset was used to determine the expression levels of *IL-1RN* following CSD in a murine model of FHM1 (Figure 1). No significant differences in *IL-1RN* levels were observed in FHM1 R192Q vs. wild-type (WT) cortex at basal condition (sham). No significant differences in *IL-1RN* expression were observed following CSD induction in WT mice as compared to sham-operated WT mice. Significantly higher levels of *IL-1RN* were observed upon CSD in FHM1 R192Q animals as compared to sham-operated mice (*p* < 0.0001) (Figure 1). 

### 3.2. Identification of Genes Functionally Related to IL-1RN

**Figure 2:**
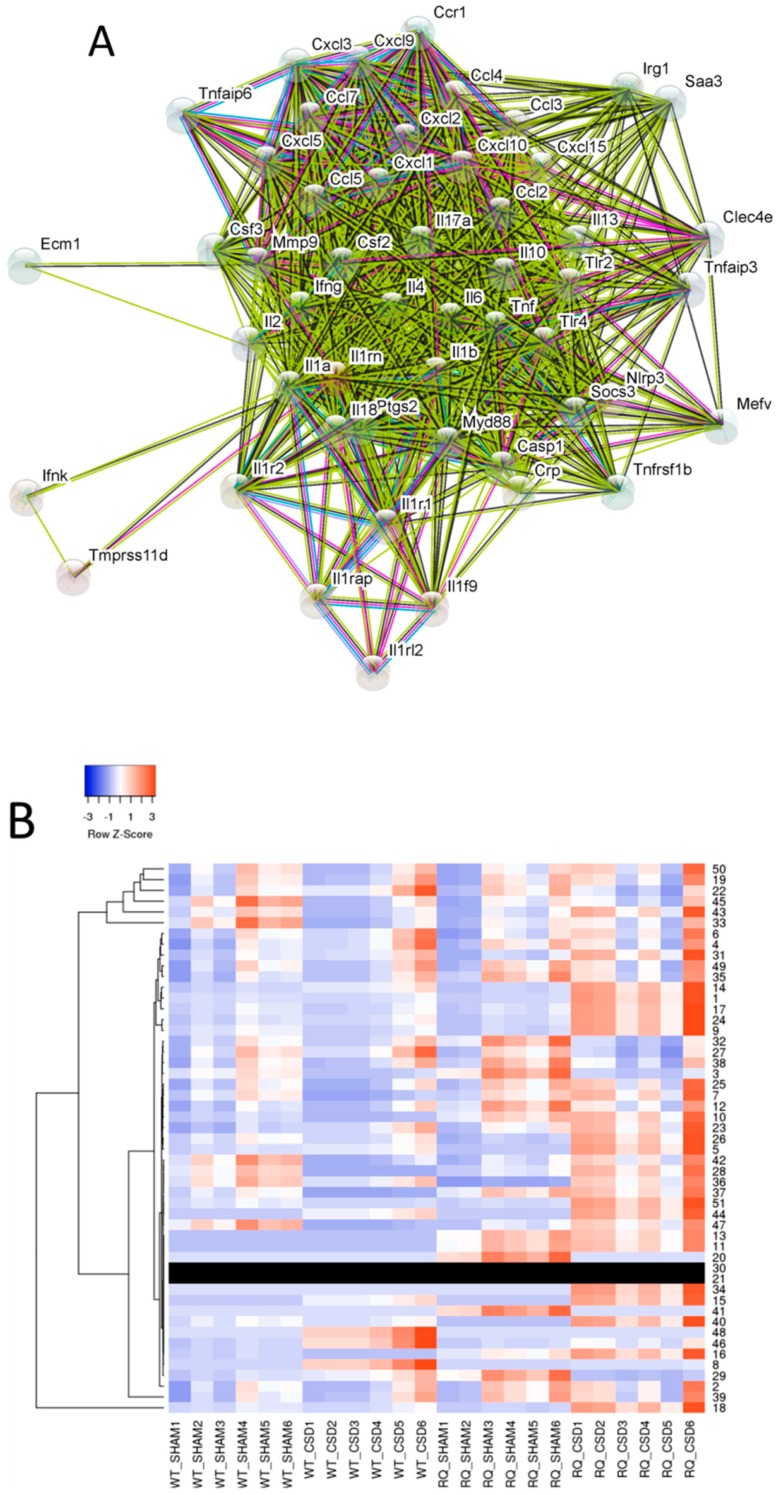
*IL-1RN* functionally-related genes. (**A**) Gene network of the *IL-1RN* functionally related genes. The edges connecting the nodes represent the interactions between genes, in terms of co-expression (black), text mining (light green), protein homology (cyan), association in curated database (light blue), and high-throughput experiments (purple). Empty nodes represent proteins of uncharacterized 3D structure, while filled nodes represent proteins with known or predicted tertiary structure. (**B**) Expression heatmap for the top correlated genes to *IL-1RN*, as determined in the GSE67933 dataset. Average linkage was used as clustering method and Euclidean distance as distance measurement. Tree branches represent the distance between genes.

We then evaluated the expression of the genes functionally related to *IL-1RN* (Figure 2A and Supplementary File 1). As shown in Figure 2 and Table 1, several of the genes identified were found to be modulated in the cortex from FHM1 R192Q mice subjected to CSD (Figure 2B). Among them, a significant upregulation of *IL-6*, *TNF*, *TLR2*, and *TLR4* could be observed in FHM1 R192Q mice upon CSD as compared to FHM1 R192Q sham-operated mice (Supplementary Table S1). Moreover, as compared to CSD-induced WT mice, FHM1 R192Q mice upon CSD expressed lower levels of *IL-18, IL-10*, *IL-2*, *IL-4*, and *IL-13*, and significantly higher levels of *IFNK*, *IL-17A*, *SOCS3*, and of several members of the chemokine/chemokine receptor family (*CCL2*, *CXCL2*, *CXCL10*, *CCL4*, *CCL7*, *CXCL1*, *CXCL3*, *CCR1*, *CXCL9*).

### 3.3. Identification of Genes Statistically Correlated to IL-1RN

**Figure 3:**
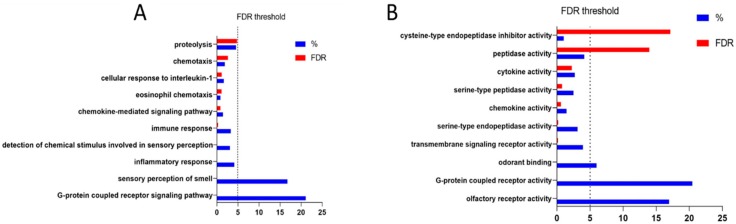
Gene ontology analysis for the genes statistically correlated to *IL-1RN* in the model of cortical spreading depression (CSD)-induced migraine. (**A**) Most enriched biological processes; (**B**) Most enriched molecular functions. FDR: False Discovery Rate

We found 927 genes statistically correlated to *IL-1RN* in CDS-induced FHM1 R192Q mice, considering as threshold a Pearson correlation coefficient (r) > 0.95 and a *p*-value < 0.05. The top 20 statistically significant correlated genes are presented in Table 1. Gene Ontology for biological processes (BPs) revealed a significant enrichment of genes involved in the “G-protein coupled receptor signaling pathway” (*p* < 0.0001), “sensory perception of smell” (*p* < 0.0001) and “inflammatory response” (*p* < 0.0001) (Figure 3A). The top enriched molecular functions (MFs) were: “olfactory receptor activity” (*p* < 0.0001), G-protein coupled receptor activity (*p* < 0.0001) and “odorant binding” (*p* < 0.0001) (Figure 3B).

## 4. Discussion

Many studies have shown a relationship between migraine and inflammation [51,52]. Neurogenic inflammation is characterized by the release of vasoactive neuropeptides from nociceptive sensory nerve terminals, including calcitonin gene-related peptide (CGRP), substance P (SP), and neurokinin A. These peptides lead to the dilatation of vessels, with increased permeability and consequent exudation of fluids, plasma proteins, leukocyte extravasation, and mast cell degranulation [51]. In particular, it has been proposed that migraine may be associated with an inflammation of the meninges, especially the dura mater. During a migraine attack, an idiopathic activation of the trigeminal sensory afferents is thought to facilitate the nociceptive transmission to the central nervous system (CNS). Accordingly, inhibition of the dural neurogenic inflammation with molecules able to inhibit the pathways involved in the activation and sensitization of trigeminovascular neurons—at both their central and peripheral perivascular nerve endings—have been tested as potential therapeutic strategies in the treatment of migraine [51].

The main current therapy for migraine is based on triptans [53], which have been shown to attenuate the release of neuropeptides and neurogenic plasma protein extravasation. These findings provide support for the validity of using animal models of neurogenic inflammation to investigate putative etiopathogenic mechanisms in migraine. 

During the interictal period (headache-free days), independent studies have demonstrated increased peripheral levels of the pro-inflammatory cytokines IL-1β, IL-6, and TNF-α, and of the chemokine IL-8. On the other hand, the levels of the anti-inflammatory cytokine IL-10 have been found to be either unaltered or reduced in migraine patients as compared to healthy controls. Moreover, during a migraine attack, the serum concentrations of IL-1β, IL-6, IL-8, and TNF-α increase, alongside the levels of IL-10; in contrast, the type 2 cytokines IL-4 and IL-5 decrease [54]. Interestingly, migraine seems to be associated with immune-inflammatory and atopic diseases sustained by both Th1- or Th2-dominant responses, such as Inflammatory Bowel Disease and asthma [54]. 

Many studies have already observed that CSD induces the upregulation of several pro-inflammatory cytokines [6,40,44,55,56,57,58]. In the present study, we evaluated the expression of *IL-1RN* during CSD in a model of FMH1 by using a publicly available deep SAGE dataset. The use of whole-genome expression data has been extensively used for the identification of novel pathogenic pathways and therapeutic targets in a variety of diseases (e.g., autoimmunity [59,60,61,62], cancer [63,64,65,66], hepatic [67,68], neurodegenerative, and infectious diseases [69]). 

The protein encoded by *IL-1RN* is a soluble factor that regulates the inflammatory response, as reported by various studies [21,22,70,71]. However, its role in migraine is still largely unexplored. In a previous study, significantly higher levels of *IL-1RN*, *TGF-β1*, and *MCP-1* in the cerebrospinal fluid of migraine patients as compared to controls were found [72].

Unlike *IL-2*, *IL-4*, *IL-10*, and *IL-13*, the expression of *IL-1RN* and *IL-6* was higher in the brains of FHM1 R192Q mutant mice than in WT mice, probably due to an alteration in Ca^2+^ ion channels. We can speculate that these molecules may exert a homeostatic role aimed at counteracting ongoing immunoinflammatory events. Even if the precise biological mechanisms by which IL-1RN production is increased is not known, it could be explained by an induction promoted by both IL-6 [73] and IFN-α [74]. Indeed, a role for the IFN pathway in migraine has been described [44]. We may speculate that compensatory mechanisms may be working constantly in migraine patients via the reciprocal regulation of pro- and anti-inflammatory factors, leading to Th1-dominant responses and the consequent effects associated with these cytokines. Moreover, we found an important increase in several members of the chemokine/chemokine receptor family in FHM1 R192Q, which could contribute to the vasodilatation and to the swelling mechanism underlying migraine and nasal congestion, which represents one of the most common symptoms [75]. 

The role of inflammation in migraine is further supported by the therapeutic effects of non-steroidal anti-inflammatory drugs (NSAIDs), currently recommended as the first-line medications for acute migraine attacks, as they improve both pain and breathing [76,77]. Recently, monoclonal antibodies targeting the CGRP pathway (i.e. fremanezumab, eptinezumab, and galcanezumab) have also been tested in both chronic and episodic migraine, and were shown to improve migraine-associated symptoms, quality of life, and disability, and to reduce monthly migraine days [78,79,80,81].

This study may set the basis for new therapeutic strategies for the treatment of migraine, such as anakinra [82]. Anakinra is a recombinant nonglycosylated analogue of the human IL-1RN which competitively blocks the binding of IL-1ß and IL-1α to the IL-1 receptors. Anakinra was approved by the Food and Drug Administration (FDA) in 2012 for chronic infantile neurological cutaneous and articular syndrome (CINCA) and in 2013 by the European Medicines Agency (EMA) for all subtypes of cryopyrin-associated periodic syndrome (CAPS). In nonhuman primates, anakinra has been shown to be able to cross the blood–brain barrier in a dose-dependent manner. Therefore, a direct anti-inflammatory action in the CNS is plausible [83]. Although only marginally related to migraine, a recent prospective, open-label, long-term study in 43 patients with severe cryopyrin-associated periodic syndromes (CAPS) demonstrated that anakinra treatment significantly decreased central nervous system inflammation and headaches in pediatric patients.

## 5. Conclusions

Little is known about the etiology and physiopathogenesis of migraine, but a large body of evidence shows a relationship with inflammation. In this paper, we show the gene expression of pro- and anti-inflammatory cytokines in a mouse migraine model. In particular, we focus on the expression of *IL-1RN*, which appears to be over-expressed after the CSD, suggesting a possible attempt to modulate the inflammatory response. This study may be the first one that allows a better understanding of the development of the disease and may aid in the search for new therapeutic strategies in migraine.

## Figures and Tables

**Table 1:** Top 20 genes statistically correlated to *IL-1RN*.

Gene	Gene Stable ID	Pearson r	95% Confidence Interval	*p*-Value	*R* Squared
Cdhr5	ENSMUSG00000025497	0.9996	0.9991 to 0.9998	<0.0001	0.9992
Gm8251	ENSMUSG00000091844	0.9996	0.9991 to 0.9998	<0.0001	0.9993
Scarna3b	ENSMUSG00000088158	0.9993	0.9983 to 0.9997	<0.0001	0.9986
Nr0b1	ENSMUSG00000025056	0.9991	0.9980 to 0.9996	<0.0001	0.9983
Lrrc15	ENSMUSG00000052316	0.9987	0.9968 to 0.9994	<0.0001	0.9973
Ifi204	ENSMUSG00000073489	0.9982	0.9959 to 0.9993	<0.0001	0.9965
Cd200r3	ENSMUSG00000036172	0.9981	0.9955 to 0.9992	<0.0001	0.9962
Gm13389	ENSMUSG00000087079	0.9977	0.9947 to 0.9990	<0.0001	0.9955
Hpx	ENSMUSG00000030895	0.9976	0.9944 to 0.9990	<0.0001	0.9953
Gm15941	ENSMUSG00000086992	0.9974	0.9940 to 0.9989	<0.0001	0.9949
Zc3h12d	ENSMUSG00000039981	0.9973	0.9938 to 0.9989	<0.0001	0.9947
Gm49339	ENSMUSG00000062593	0.9973	0.9936 to 0.9988	<0.0001	0.9945
Klre1	ENSMUSG00000050241	0.9971	0.9933 to 0.9988	<0.0001	0.9943
Klk9	ENSMUSG00000047884	0.9966	0.9921 to 0.9986	<0.0001	0.9933
Gm22486	ENSMUSG00000080465	0.9966	0.9921 to 0.9986	<0.0001	0.9933
Abo	ENSMUSG00000015787	0.9965	0.9919 to 0.9985	<0.0001	0.9931
Cnga3	ENSMUSG00000026114	0.9965	0.9917 to 0.9985	<0.0001	0.9929
Ccl4	ENSMUSG00000018930	0.9960	0.9906 to 0.9983	<0.0001	0.9920
Snord66	ENSMUSG00000077239	0.9958	0.9901 to 0.9982	<0.0001	0.9916
Gm13429	ENSMUSG00000085141	0.9957	0.9898 to 0.9982	<0.0001	0.9913

## Supplementary Materials

The following are available online at https://www.mdpi.com/2076-3425/9/7/172/s1, File 1: Genes functionally related to *IL1RN*, Table S1: Multiple comparison.

## Sample Availability

Data are available from the Gene Expression Ombnibus Database.
