# Supplementary material for: Upregulation of IL-1 Receptor Antagonist in a Mouse Model of Migraine
Source: Brain Sci. 2019 Jul 19;9(7):172. doi: 10.3390/brainsci9070172 (PMC6680509; doi:10.3390/brainsci9070172)
Supplement: Supplementary file 1 [file brainsci-09-00172-s001.zip › Supplementary Table 1.docx]

| **Multiple comparison** | | | | | | | |
| --- | --- | --- | --- | --- | --- | --- | --- |
| Bonferroni | | | | | | | |
| Dependent variable |  |  | Mean difference | std.error | Sign. | CI 95% | |
|  |  |  |  |  |  | Limit inferior | Limit superior |
| IL-1rn | WT_SHAM | WT_CSD | -1,913 | 7,034 | 1,000 | -22,50 | 18,67 |
|  |  | RQ_SHAM | 2,238 | 7,034 | 1,000 | -18,35 | 22,83 |
|  |  | RQ_CSD | -48,952^*^ | 7,034 | ,000 | -69,54 | -28,36 |
|  | WT_CSD | WT_SHAM | 1,913 | 7,034 | 1,000 | -18,67 | 22,50 |
|  |  | RQ_SHAM | 4,152 | 7,034 | 1,000 | -16,44 | 24,74 |
|  |  | RQ_CSD | -47,039^*^ | 7,034 | ,000 | -67,63 | -26,45 |
|  | RQ_SHAM | WT_SHAM | -2,238 | 7,034 | 1,000 | -22,83 | 18,35 |
|  |  | WT_CSD | -4,152 | 7,034 | 1,000 | -24,74 | 16,44 |
|  |  | RQ_CSD | -51,190^*^ | 7,034 | ,000 | -71,78 | -30,60 |
|  | RQ_CSD | WT_SHAM | 48,952^*^ | 7,034 | ,000 | 28,36 | 69,54 |
|  |  | WT_CSD | 47,039^*^ | 7,034 | ,000 | 26,45 | 67,63 |
|  |  | RQ_SHAM | 51,190^*^ | 7,034 | ,000 | 30,60 | 71,78 |
| IL1R1 | WT_SHAM | WT_CSD | -,084 | 4,498 | 1,000 | -13,25 | 13,08 |
|  |  | RQ_SHAM | -4,850 | 4,498 | 1,000 | -18,02 | 8,32 |
|  |  | RQ_CSD | -5,981 | 4,498 | 1,000 | -19,15 | 7,19 |
|  | WT_CSD | WT_SHAM | ,084 | 4,498 | 1,000 | -13,08 | 13,25 |
|  |  | RQ_SHAM | -4,766 | 4,498 | 1,000 | -17,93 | 8,40 |
|  |  | RQ_CSD | -5,897 | 4,498 | 1,000 | -19,06 | 7,27 |
|  | RQ_SHAM | WT_SHAM | 4,850 | 4,498 | 1,000 | -8,32 | 18,02 |
|  |  | WT_CSD | 4,766 | 4,498 | 1,000 | -8,40 | 17,93 |
|  |  | RQ_CSD | -1,130 | 4,498 | 1,000 | -14,30 | 12,04 |
|  | RQ_CSD | WT_SHAM | 5,981 | 4,498 | 1,000 | -7,19 | 19,15 |
|  |  | WT_CSD | 5,897 | 4,498 | 1,000 | -7,27 | 19,06 |
|  |  | RQ_SHAM | 1,130 | 4,498 | 1,000 | -12,04 | 14,30 |
| IL1R2 | WT_SHAM | WT_CSD | 5,736 | 2,098 | ,077 | -,40 | 11,88 |
|  |  | RQ_SHAM | -7,997^*^ | 2,098 | ,007 | -14,14 | -1,86 |
|  |  | RQ_CSD | 5,108 | 2,098 | ,146 | -1,03 | 11,25 |
|  | WT_CSD | WT_SHAM | -5,736 | 2,098 | ,077 | -11,88 | ,40 |
|  |  | RQ_SHAM | -13,733^*^ | 2,098 | ,000 | -19,87 | -7,59 |
|  |  | RQ_CSD | -,628 | 2,098 | 1,000 | -6,77 | 5,51 |
|  | RQ_SHAM | WT_SHAM | 7,997^*^ | 2,098 | ,007 | 1,86 | 14,14 |
|  |  | WT_CSD | 13,733^*^ | 2,098 | ,000 | 7,59 | 19,87 |
|  |  | RQ_CSD | 13,105^*^ | 2,098 | ,000 | 6,96 | 19,25 |
|  | RQ_CSD | WT_SHAM | -5,108 | 2,098 | ,146 | -11,25 | 1,03 |
|  |  | WT_CSD | ,628 | 2,098 | 1,000 | -5,51 | 6,77 |
|  |  | RQ_SHAM | -13,105^*^ | 2,098 | ,000 | -19,25 | -6,96 |
| IL1a | WT_SHAM | WT_CSD | -11,564 | 7,177 | ,737 | -32,57 | 9,44 |
|  |  | RQ_SHAM | -8,269 | 7,177 | 1,000 | -29,28 | 12,74 |
|  |  | RQ_CSD | -8,765 | 7,177 | 1,000 | -29,77 | 12,24 |
|  | WT_CSD | WT_SHAM | 11,564 | 7,177 | ,737 | -9,44 | 32,57 |
|  |  | RQ_SHAM | 3,295 | 7,177 | 1,000 | -17,71 | 24,30 |
|  |  | RQ_CSD | 2,799 | 7,177 | 1,000 | -18,21 | 23,81 |
|  | RQ_SHAM | WT_SHAM | 8,269 | 7,177 | 1,000 | -12,74 | 29,28 |
|  |  | WT_CSD | -3,295 | 7,177 | 1,000 | -24,30 | 17,71 |
|  |  | RQ_CSD | -,496 | 7,177 | 1,000 | -21,50 | 20,51 |
|  | RQ_CSD | WT_SHAM | 8,765 | 7,177 | 1,000 | -12,24 | 29,77 |
|  |  | WT_CSD | -2,799 | 7,177 | 1,000 | -23,81 | 18,21 |
|  |  | RQ_SHAM | ,496 | 7,177 | 1,000 | -20,51 | 21,50 |
| IL6 | WT_SHAM | WT_CSD | -2,609 | 2,009 | 1,000 | -8,49 | 3,27 |
|  |  | RQ_SHAM | 1,718 | 2,009 | 1,000 | -4,16 | 7,60 |
|  |  | RQ_CSD | -13,229^*^ | 2,009 | ,000 | -19,11 | -7,35 |
|  | WT_CSD | WT_SHAM | 2,609 | 2,009 | 1,000 | -3,27 | 8,49 |
|  |  | RQ_SHAM | 4,327 | 2,009 | ,262 | -1,55 | 10,21 |
|  |  | RQ_CSD | -10,620^*^ | 2,009 | ,000 | -16,50 | -4,74 |
|  | RQ_SHAM | WT_SHAM | -1,718 | 2,009 | 1,000 | -7,60 | 4,16 |
|  |  | WT_CSD | -4,327 | 2,009 | ,262 | -10,21 | 1,55 |
|  |  | RQ_CSD | -14,947^*^ | 2,009 | ,000 | -20,83 | -9,07 |
|  | RQ_CSD | WT_SHAM | 13,229^*^ | 2,009 | ,000 | 7,35 | 19,11 |
|  |  | WT_CSD | 10,620^*^ | 2,009 | ,000 | 4,74 | 16,50 |
|  |  | RQ_SHAM | 14,947^*^ | 2,009 | ,000 | 9,07 | 20,83 |
| Tnfrsf1b | WT_SHAM | WT_CSD | -8,534 | 8,348 | 1,000 | -32,97 | 15,90 |
|  |  | RQ_SHAM | 3,302 | 8,348 | 1,000 | -21,13 | 27,74 |
|  |  | RQ_CSD | -11,636 | 8,348 | 1,000 | -36,07 | 12,80 |
|  | WT_CSD | WT_SHAM | 8,534 | 8,348 | 1,000 | -15,90 | 32,97 |
|  |  | RQ_SHAM | 11,836 | 8,348 | 1,000 | -12,60 | 36,27 |
|  |  | RQ_CSD | -3,102 | 8,348 | 1,000 | -27,54 | 21,33 |
|  | RQ_SHAM | WT_SHAM | -3,302 | 8,348 | 1,000 | -27,74 | 21,13 |
|  |  | WT_CSD | -11,836 | 8,348 | 1,000 | -36,27 | 12,60 |
|  |  | RQ_CSD | -14,938 | 8,348 | ,532 | -39,37 | 9,50 |
|  | RQ_CSD | WT_SHAM | 11,636 | 8,348 | 1,000 | -12,80 | 36,07 |
|  |  | WT_CSD | 3,102 | 8,348 | 1,000 | -21,33 | 27,54 |
|  |  | RQ_SHAM | 14,938 | 8,348 | ,532 | -9,50 | 39,37 |
| TNF | WT_SHAM | WT_CSD | 4,167 | 1,730 | ,155 | -,90 | 9,23 |
|  |  | RQ_SHAM | -,548 | 1,730 | 1,000 | -5,61 | 4,52 |
|  |  | RQ_CSD | -4,108 | 1,730 | ,166 | -9,17 | ,96 |
|  | WT_CSD | WT_SHAM | -4,167 | 1,730 | ,155 | -9,23 | ,90 |
|  |  | RQ_SHAM | -4,715 | 1,730 | ,078 | -9,78 | ,35 |
|  |  | RQ_CSD | -8,274^*^ | 1,730 | ,001 | -13,34 | -3,21 |
|  | RQ_SHAM | WT_SHAM | ,548 | 1,730 | 1,000 | -4,52 | 5,61 |
|  |  | WT_CSD | 4,715 | 1,730 | ,078 | -,35 | 9,78 |
|  |  | RQ_CSD | -3,559 | 1,730 | ,318 | -8,62 | 1,51 |
|  | RQ_CSD | WT_SHAM | 4,108 | 1,730 | ,166 | -,96 | 9,17 |
|  |  | WT_CSD | 8,274^*^ | 1,730 | ,001 | 3,21 | 13,34 |
|  |  | RQ_SHAM | 3,559 | 1,730 | ,318 | -1,51 | 8,62 |
| IL10 | WT_SHAM | WT_CSD | -2,596^*^ | ,357 | ,000 | -3,64 | -1,55 |
|  |  | RQ_SHAM | ,000 | ,357 | 1,000 | -1,05 | 1,05 |
|  |  | RQ_CSD | -,164 | ,357 | 1,000 | -1,21 | ,88 |
|  | WT_CSD | WT_SHAM | 2,596^*^ | ,357 | ,000 | 1,55 | 3,64 |
|  |  | RQ_SHAM | 2,596^*^ | ,357 | ,000 | 1,55 | 3,64 |
|  |  | RQ_CSD | 2,432^*^ | ,357 | ,000 | 1,39 | 3,48 |
|  | RQ_SHAM | WT_SHAM | ,000 | ,357 | 1,000 | -1,05 | 1,05 |
|  |  | WT_CSD | -2,596^*^ | ,357 | ,000 | -3,64 | -1,55 |
|  |  | RQ_CSD | -,164 | ,357 | 1,000 | -1,21 | ,88 |
|  | RQ_CSD | WT_SHAM | ,164 | ,357 | 1,000 | -,88 | 1,21 |
|  |  | WT_CSD | -2,432^*^ | ,357 | ,000 | -3,48 | -1,39 |
|  |  | RQ_SHAM | ,164 | ,357 | 1,000 | -,88 | 1,21 |
| Ccl2 | WT_SHAM | WT_CSD | 8,812 | 13,495 | 1,000 | -30,69 | 48,31 |
|  |  | RQ_SHAM | 13,526 | 13,495 | 1,000 | -25,98 | 53,03 |
|  |  | RQ_CSD | -71,466^*^ | 13,495 | ,000 | -110,97 | -31,96 |
|  | WT_CSD | WT_SHAM | -8,812 | 13,495 | 1,000 | -48,31 | 30,69 |
|  |  | RQ_SHAM | 4,714 | 13,495 | 1,000 | -34,79 | 44,22 |
|  |  | RQ_CSD | -80,278^*^ | 13,495 | ,000 | -119,78 | -40,78 |
|  | RQ_SHAM | WT_SHAM | -13,526 | 13,495 | 1,000 | -53,03 | 25,98 |
|  |  | WT_CSD | -4,714 | 13,495 | 1,000 | -44,22 | 34,79 |
|  |  | RQ_CSD | -84,992^*^ | 13,495 | ,000 | -124,49 | -45,49 |
|  | RQ_CSD | WT_SHAM | 71,466^*^ | 13,495 | ,000 | 31,96 | 110,97 |
|  |  | WT_CSD | 80,278^*^ | 13,495 | ,000 | 40,78 | 119,78 |
|  |  | RQ_SHAM | 84,992^*^ | 13,495 | ,000 | 45,49 | 124,49 |
| Cxcl2 | WT_SHAM | WT_CSD | 1,276 | 1,331 | 1,000 | -2,62 | 5,17 |
|  |  | RQ_SHAM | -4,318^*^ | 1,331 | ,024 | -8,21 | -,42 |
|  |  | RQ_CSD | -6,758^*^ | 1,331 | ,000 | -10,65 | -2,86 |
|  | WT_CSD | WT_SHAM | -1,276 | 1,331 | 1,000 | -5,17 | 2,62 |
|  |  | RQ_SHAM | -5,594^*^ | 1,331 | ,003 | -9,49 | -1,70 |
|  |  | RQ_CSD | -8,034^*^ | 1,331 | ,000 | -11,93 | -4,14 |
|  | RQ_SHAM | WT_SHAM | 4,318^*^ | 1,331 | ,024 | ,42 | 8,21 |
|  |  | WT_CSD | 5,594^*^ | 1,331 | ,003 | 1,70 | 9,49 |
|  |  | RQ_CSD | -2,440 | 1,331 | ,491 | -6,34 | 1,46 |
|  | RQ_CSD | WT_SHAM | 6,758^*^ | 1,331 | ,000 | 2,86 | 10,65 |
|  |  | WT_CSD | 8,034^*^ | 1,331 | ,000 | 4,14 | 11,93 |
|  |  | RQ_SHAM | 2,440 | 1,331 | ,491 | -1,46 | 6,34 |
| Tmprss11d | WT_SHAM | WT_CSD | ,000 | ,027 | 1,000 | -,08 | ,08 |
|  |  | RQ_SHAM | -,152^*^ | ,027 | ,000 | -,23 | -,07 |
|  |  | RQ_CSD | -,168^*^ | ,027 | ,000 | -,25 | -,09 |
|  | WT_CSD | WT_SHAM | ,000 | ,027 | 1,000 | -,08 | ,08 |
|  |  | RQ_SHAM | -,152^*^ | ,027 | ,000 | -,23 | -,07 |
|  |  | RQ_CSD | -,168^*^ | ,027 | ,000 | -,25 | -,09 |
|  | RQ_SHAM | WT_SHAM | ,152^*^ | ,027 | ,000 | ,07 | ,23 |
|  |  | WT_CSD | ,152^*^ | ,027 | ,000 | ,07 | ,23 |
|  |  | RQ_CSD | -,016 | ,027 | 1,000 | -,10 | ,06 |
|  | RQ_CSD | WT_SHAM | ,168^*^ | ,027 | ,000 | ,09 | ,25 |
|  |  | WT_CSD | ,168^*^ | ,027 | ,000 | ,09 | ,25 |
|  |  | RQ_SHAM | ,016 | ,027 | 1,000 | -,06 | ,10 |
| Nlrp3 | WT_SHAM | WT_CSD | -,411 | 1,540 | 1,000 | -4,92 | 4,10 |
|  |  | RQ_SHAM | -4,410 | 1,540 | ,058 | -8,92 | ,10 |
|  |  | RQ_CSD | -2,379 | 1,540 | ,829 | -6,89 | 2,13 |
|  | WT_CSD | WT_SHAM | ,411 | 1,540 | 1,000 | -4,10 | 4,92 |
|  |  | RQ_SHAM | -3,999 | 1,540 | ,104 | -8,51 | ,51 |
|  |  | RQ_CSD | -1,968 | 1,540 | 1,000 | -6,48 | 2,54 |
|  | RQ_SHAM | WT_SHAM | 4,410 | 1,540 | ,058 | -,10 | 8,92 |
|  |  | WT_CSD | 3,999 | 1,540 | ,104 | -,51 | 8,51 |
|  |  | RQ_CSD | 2,031 | 1,540 | 1,000 | -2,48 | 6,54 |
|  | RQ_CSD | WT_SHAM | 2,379 | 1,540 | ,829 | -2,13 | 6,89 |
|  |  | WT_CSD | 1,968 | 1,540 | 1,000 | -2,54 | 6,48 |
|  |  | RQ_SHAM | -2,031 | 1,540 | 1,000 | -6,54 | 2,48 |
| IL1f9 | WT_SHAM | WT_CSD | ,000 | ,028 | 1,000 | -,08 | ,08 |
|  |  | RQ_SHAM | -,158^*^ | ,028 | ,000 | -,24 | -,08 |
|  |  | RQ_CSD | -,165^*^ | ,028 | ,000 | -,25 | -,08 |
|  | WT_CSD | WT_SHAM | ,000 | ,028 | 1,000 | -,08 | ,08 |
|  |  | RQ_SHAM | -,158^*^ | ,028 | ,000 | -,24 | -,08 |
|  |  | RQ_CSD | -,165^*^ | ,028 | ,000 | -,25 | -,08 |
|  | RQ_SHAM | WT_SHAM | ,158^*^ | ,028 | ,000 | ,08 | ,24 |
|  |  | WT_CSD | ,158^*^ | ,028 | ,000 | ,08 | ,24 |
|  |  | RQ_CSD | -,007 | ,028 | 1,000 | -,09 | ,07 |
|  | RQ_CSD | WT_SHAM | ,165^*^ | ,028 | ,000 | ,08 | ,25 |
|  |  | WT_CSD | ,165^*^ | ,028 | ,000 | ,08 | ,25 |
|  |  | RQ_SHAM | ,007 | ,028 | 1,000 | -,07 | ,09 |
| Cxcl10 | WT_SHAM | WT_CSD | -15,195 | 8,270 | ,486 | -39,40 | 9,01 |
|  |  | RQ_SHAM | ,563 | 8,270 | 1,000 | -23,64 | 24,77 |
|  |  | RQ_CSD | -52,672^*^ | 8,270 | ,000 | -76,88 | -28,46 |
|  | WT_CSD | WT_SHAM | 15,195 | 8,270 | ,486 | -9,01 | 39,40 |
|  |  | RQ_SHAM | 15,758 | 8,270 | ,427 | -8,45 | 39,97 |
|  |  | RQ_CSD | -37,477^*^ | 8,270 | ,001 | -61,69 | -13,27 |
|  | RQ_SHAM | WT_SHAM | -,563 | 8,270 | 1,000 | -24,77 | 23,64 |
|  |  | WT_CSD | -15,758 | 8,270 | ,427 | -39,97 | 8,45 |
|  |  | RQ_CSD | -53,235^*^ | 8,270 | ,000 | -77,44 | -29,03 |
|  | RQ_CSD | WT_SHAM | 52,672^*^ | 8,270 | ,000 | 28,46 | 76,88 |
|  |  | WT_CSD | 37,477^*^ | 8,270 | ,001 | 13,27 | 61,69 |
|  |  | RQ_SHAM | 53,235^*^ | 8,270 | ,000 | 29,03 | 77,44 |
| Il1rl2 | WT_SHAM | WT_CSD | -,190 | ,065 | ,052 | -,38 | ,00 |
|  |  | RQ_SHAM | ,000 | ,065 | 1,000 | -,19 | ,19 |
|  |  | RQ_CSD | -,484^*^ | ,065 | ,000 | -,67 | -,29 |
|  | WT_CSD | WT_SHAM | ,190 | ,065 | ,052 | ,00 | ,38 |
|  |  | RQ_SHAM | ,190 | ,065 | ,052 | ,00 | ,38 |
|  |  | RQ_CSD | -,294^*^ | ,065 | ,001 | -,49 | -,10 |
|  | RQ_SHAM | WT_SHAM | ,000 | ,065 | 1,000 | -,19 | ,19 |
|  |  | WT_CSD | -,190 | ,065 | ,052 | -,38 | ,00 |
|  |  | RQ_CSD | -,484^*^ | ,065 | ,000 | -,67 | -,29 |
|  | RQ_CSD | WT_SHAM | ,484^*^ | ,065 | ,000 | ,29 | ,67 |
|  |  | WT_CSD | ,294^*^ | ,065 | ,001 | ,10 | ,49 |
|  |  | RQ_SHAM | ,484^*^ | ,065 | ,000 | ,29 | ,67 |
| Ifnk | WT_SHAM | WT_CSD | ,208 | ,229 | 1,000 | -,46 | ,88 |
|  |  | RQ_SHAM | -,498 | ,229 | ,251 | -1,17 | ,17 |
|  |  | RQ_CSD | -1,508^*^ | ,229 | ,000 | -2,18 | -,84 |
|  | WT_CSD | WT_SHAM | -,208 | ,229 | 1,000 | -,88 | ,46 |
|  |  | RQ_SHAM | -,706^*^ | ,229 | ,035 | -1,38 | -,04 |
|  |  | RQ_CSD | -1,717^*^ | ,229 | ,000 | -2,39 | -1,05 |
|  | RQ_SHAM | WT_SHAM | ,498 | ,229 | ,251 | -,17 | 1,17 |
|  |  | WT_CSD | ,706^*^ | ,229 | ,035 | ,04 | 1,38 |
|  |  | RQ_CSD | -1,010^*^ | ,229 | ,002 | -1,68 | -,34 |
|  | RQ_CSD | WT_SHAM | 1,508^*^ | ,229 | ,000 | ,84 | 2,18 |
|  |  | WT_CSD | 1,717^*^ | ,229 | ,000 | 1,05 | 2,39 |
|  |  | RQ_SHAM | 1,010^*^ | ,229 | ,002 | ,34 | 1,68 |
| Ccl3 | WT_SHAM | WT_CSD | -,355 | 9,437 | 1,000 | -27,98 | 27,27 |
|  |  | RQ_SHAM | 2,509 | 9,437 | 1,000 | -25,12 | 30,13 |
|  |  | RQ_CSD | -51,543^*^ | 9,437 | ,000 | -79,17 | -23,92 |
|  | WT_CSD | WT_SHAM | ,355 | 9,437 | 1,000 | -27,27 | 27,98 |
|  |  | RQ_SHAM | 2,864 | 9,437 | 1,000 | -24,76 | 30,49 |
|  |  | RQ_CSD | -51,188^*^ | 9,437 | ,000 | -78,81 | -23,56 |
|  | RQ_SHAM | WT_SHAM | -2,509 | 9,437 | 1,000 | -30,13 | 25,12 |
|  |  | WT_CSD | -2,864 | 9,437 | 1,000 | -30,49 | 24,76 |
|  |  | RQ_CSD | -54,052^*^ | 9,437 | ,000 | -81,68 | -26,43 |
|  | RQ_CSD | WT_SHAM | 51,543^*^ | 9,437 | ,000 | 23,92 | 79,17 |
|  |  | WT_CSD | 51,188^*^ | 9,437 | ,000 | 23,56 | 78,81 |
|  |  | RQ_SHAM | 54,052^*^ | 9,437 | ,000 | 26,43 | 81,68 |
| Ccl4 | WT_SHAM | WT_CSD | -9,639 | 127,667 | 1,000 | -383,34 | 364,06 |
|  |  | RQ_SHAM | 113,616 | 127,667 | 1,000 | -260,08 | 487,31 |
|  |  | RQ_CSD | -780,032^*^ | 127,667 | ,000 | -1153,73 | -406,34 |
|  | WT_CSD | WT_SHAM | 9,639 | 127,667 | 1,000 | -364,06 | 383,34 |
|  |  | RQ_SHAM | 123,255 | 127,667 | 1,000 | -250,44 | 496,95 |
|  |  | RQ_CSD | -770,393^*^ | 127,667 | ,000 | -1144,09 | -396,70 |
|  | RQ_SHAM | WT_SHAM | -113,616 | 127,667 | 1,000 | -487,31 | 260,08 |
|  |  | WT_CSD | -123,255 | 127,667 | 1,000 | -496,95 | 250,44 |
|  |  | RQ_CSD | -893,648^*^ | 127,667 | ,000 | -1267,34 | -519,95 |
|  | RQ_CSD | WT_SHAM | 780,032^*^ | 127,667 | ,000 | 406,34 | 1153,73 |
|  |  | WT_CSD | 770,393^*^ | 127,667 | ,000 | 396,70 | 1144,09 |
|  |  | RQ_SHAM | 893,648^*^ | 127,667 | ,000 | 519,95 | 1267,34 |
| Il1rap | WT_SHAM | WT_CSD | -4,329 | 78,348 | 1,000 | -233,66 | 225,01 |
|  |  | RQ_SHAM | -15,056 | 78,348 | 1,000 | -244,39 | 214,28 |
|  |  | RQ_CSD | -51,653 | 78,348 | 1,000 | -280,99 | 177,68 |
|  | WT_CSD | WT_SHAM | 4,329 | 78,348 | 1,000 | -225,01 | 233,66 |
|  |  | RQ_SHAM | -10,727 | 78,348 | 1,000 | -240,06 | 218,61 |
|  |  | RQ_CSD | -47,323 | 78,348 | 1,000 | -276,66 | 182,01 |
|  | RQ_SHAM | WT_SHAM | 15,056 | 78,348 | 1,000 | -214,28 | 244,39 |
|  |  | WT_CSD | 10,727 | 78,348 | 1,000 | -218,61 | 240,06 |
|  |  | RQ_CSD | -36,597 | 78,348 | 1,000 | -265,93 | 192,74 |
|  | RQ_CSD | WT_SHAM | 51,653 | 78,348 | 1,000 | -177,68 | 280,99 |
|  |  | WT_CSD | 47,323 | 78,348 | 1,000 | -182,01 | 276,66 |
|  |  | RQ_SHAM | 36,597 | 78,348 | 1,000 | -192,74 | 265,93 |
| Cxcl15 | WT_SHAM | WT_CSD | ,000 | ,018 | 1,000 | -,05 | ,05 |
|  |  | RQ_SHAM | -,152^*^ | ,018 | ,000 | -,20 | -,10 |
|  |  | RQ_CSD | ,000 | ,018 | 1,000 | -,05 | ,05 |
|  | WT_CSD | WT_SHAM | ,000 | ,018 | 1,000 | -,05 | ,05 |
|  |  | RQ_SHAM | -,152^*^ | ,018 | ,000 | -,20 | -,10 |
|  |  | RQ_CSD | ,000 | ,018 | 1,000 | -,05 | ,05 |
|  | RQ_SHAM | WT_SHAM | ,152^*^ | ,018 | ,000 | ,10 | ,20 |
|  |  | WT_CSD | ,152^*^ | ,018 | ,000 | ,10 | ,20 |
|  |  | RQ_CSD | ,152^*^ | ,018 | ,000 | ,10 | ,20 |
|  | RQ_CSD | WT_SHAM | ,000 | ,018 | 1,000 | -,05 | ,05 |
|  |  | WT_CSD | ,000 | ,018 | 1,000 | -,05 | ,05 |
|  |  | RQ_SHAM | -,152^*^ | ,018 | ,000 | -,20 | -,10 |
| Il18 | WT_SHAM | WT_CSD | -103,499 | 66,240 | ,803 | -297,39 | 90,39 |
|  |  | RQ_SHAM | -51,040 | 66,240 | 1,000 | -244,93 | 142,85 |
|  |  | RQ_CSD | 27,004 | 66,240 | 1,000 | -166,89 | 220,90 |
|  | WT_CSD | WT_SHAM | 103,499 | 66,240 | ,803 | -90,39 | 297,39 |
|  |  | RQ_SHAM | 52,459 | 66,240 | 1,000 | -141,43 | 246,35 |
|  |  | RQ_CSD | 130,503 | 66,240 | ,377 | -63,39 | 324,40 |
|  | RQ_SHAM | WT_SHAM | 51,040 | 66,240 | 1,000 | -142,85 | 244,93 |
|  |  | WT_CSD | -52,459 | 66,240 | 1,000 | -246,35 | 141,43 |
|  |  | RQ_CSD | 78,044 | 66,240 | 1,000 | -115,85 | 271,94 |
|  | RQ_CSD | WT_SHAM | -27,004 | 66,240 | 1,000 | -220,90 | 166,89 |
|  |  | WT_CSD | -130,503 | 66,240 | ,377 | -324,40 | 63,39 |
|  |  | RQ_SHAM | -78,044 | 66,240 | 1,000 | -271,94 | 115,85 |
| Ccl5 | WT_SHAM | WT_CSD | -4,158 | 2,267 | ,489 | -10,79 | 2,48 |
|  |  | RQ_SHAM | -2,866 | 2,267 | 1,000 | -9,50 | 3,77 |
|  |  | RQ_CSD | -9,109^*^ | 2,267 | ,004 | -15,74 | -2,47 |
|  | WT_CSD | WT_SHAM | 4,158 | 2,267 | ,489 | -2,48 | 10,79 |
|  |  | RQ_SHAM | 1,292 | 2,267 | 1,000 | -5,34 | 7,93 |
|  |  | RQ_CSD | -4,950 | 2,267 | ,246 | -11,58 | 1,68 |
|  | RQ_SHAM | WT_SHAM | 2,866 | 2,267 | 1,000 | -3,77 | 9,50 |
|  |  | WT_CSD | -1,292 | 2,267 | 1,000 | -7,93 | 5,34 |
|  |  | RQ_CSD | -6,243 | 2,267 | ,073 | -12,88 | ,39 |
|  | RQ_CSD | WT_SHAM | 9,109^*^ | 2,267 | ,004 | 2,47 | 15,74 |
|  |  | WT_CSD | 4,950 | 2,267 | ,246 | -1,68 | 11,58 |
|  |  | RQ_SHAM | 6,243 | 2,267 | ,073 | -,39 | 12,88 |
| Ccl7 | WT_SHAM | WT_CSD | -7,387 | 14,125 | 1,000 | -48,73 | 33,96 |
|  |  | RQ_SHAM | 3,536 | 14,125 | 1,000 | -37,81 | 44,88 |
|  |  | RQ_CSD | -80,715^*^ | 14,125 | ,000 | -122,06 | -39,37 |
|  | WT_CSD | WT_SHAM | 7,387 | 14,125 | 1,000 | -33,96 | 48,73 |
|  |  | RQ_SHAM | 10,924 | 14,125 | 1,000 | -30,42 | 52,27 |
|  |  | RQ_CSD | -73,327^*^ | 14,125 | ,000 | -114,67 | -31,98 |
|  | RQ_SHAM | WT_SHAM | -3,536 | 14,125 | 1,000 | -44,88 | 37,81 |
|  |  | WT_CSD | -10,924 | 14,125 | 1,000 | -52,27 | 30,42 |
|  |  | RQ_CSD | -84,251^*^ | 14,125 | ,000 | -125,60 | -42,90 |
|  | RQ_CSD | WT_SHAM | 80,715^*^ | 14,125 | ,000 | 39,37 | 122,06 |
|  |  | WT_CSD | 73,327^*^ | 14,125 | ,000 | 31,98 | 114,67 |
|  |  | RQ_SHAM | 84,251^*^ | 14,125 | ,000 | 42,90 | 125,60 |
| Il1b | WT_SHAM | WT_CSD | ,761 | 1,848 | 1,000 | -4,65 | 6,17 |
|  |  | RQ_SHAM | -2,023 | 1,848 | 1,000 | -7,43 | 3,39 |
|  |  | RQ_CSD | -3,451 | 1,848 | ,459 | -8,86 | 1,96 |
|  | WT_CSD | WT_SHAM | -,761 | 1,848 | 1,000 | -6,17 | 4,65 |
|  |  | RQ_SHAM | -2,783 | 1,848 | ,886 | -8,19 | 2,63 |
|  |  | RQ_CSD | -4,212 | 1,848 | ,203 | -9,62 | 1,20 |
|  | RQ_SHAM | WT_SHAM | 2,023 | 1,848 | 1,000 | -3,39 | 7,43 |
|  |  | WT_CSD | 2,783 | 1,848 | ,886 | -2,63 | 8,19 |
|  |  | RQ_CSD | -1,429 | 1,848 | 1,000 | -6,84 | 3,98 |
|  | RQ_CSD | WT_SHAM | 3,451 | 1,848 | ,459 | -1,96 | 8,86 |
|  |  | WT_CSD | 4,212 | 1,848 | ,203 | -1,20 | 9,62 |
|  |  | RQ_SHAM | 1,429 | 1,848 | 1,000 | -3,98 | 6,84 |
| Cxcl1 | WT_SHAM | WT_CSD | ,333 | 1,621 | 1,000 | -4,41 | 5,08 |
|  |  | RQ_SHAM | 2,683 | 1,621 | ,681 | -2,06 | 7,43 |
|  |  | RQ_CSD | -6,414^*^ | 1,621 | ,005 | -11,16 | -1,67 |
|  | WT_CSD | WT_SHAM | -,333 | 1,621 | 1,000 | -5,08 | 4,41 |
|  |  | RQ_SHAM | 2,350 | 1,621 | ,976 | -2,40 | 7,09 |
|  |  | RQ_CSD | -6,747^*^ | 1,621 | ,003 | -11,49 | -2,00 |
|  | RQ_SHAM | WT_SHAM | -2,683 | 1,621 | ,681 | -7,43 | 2,06 |
|  |  | WT_CSD | -2,350 | 1,621 | ,976 | -7,09 | 2,40 |
|  |  | RQ_CSD | -9,097^*^ | 1,621 | ,000 | -13,84 | -4,35 |
|  | RQ_CSD | WT_SHAM | 6,414^*^ | 1,621 | ,005 | 1,67 | 11,16 |
|  |  | WT_CSD | 6,747^*^ | 1,621 | ,003 | 2,00 | 11,49 |
|  |  | RQ_SHAM | 9,097^*^ | 1,621 | ,000 | 4,35 | 13,84 |
| Cxcl5 | WT_SHAM | WT_CSD | -1,279 | 2,148 | 1,000 | -7,57 | 5,01 |
|  |  | RQ_SHAM | -,076 | 2,148 | 1,000 | -6,36 | 6,21 |
|  |  | RQ_CSD | 2,906 | 2,148 | 1,000 | -3,38 | 9,19 |
|  | WT_CSD | WT_SHAM | 1,279 | 2,148 | 1,000 | -5,01 | 7,57 |
|  |  | RQ_SHAM | 1,203 | 2,148 | 1,000 | -5,09 | 7,49 |
|  |  | RQ_CSD | 4,186 | 2,148 | ,393 | -2,10 | 10,47 |
|  | RQ_SHAM | WT_SHAM | ,076 | 2,148 | 1,000 | -6,21 | 6,36 |
|  |  | WT_CSD | -1,203 | 2,148 | 1,000 | -7,49 | 5,09 |
|  |  | RQ_CSD | 2,983 | 2,148 | 1,000 | -3,31 | 9,27 |
|  | RQ_CSD | WT_SHAM | -2,906 | 2,148 | 1,000 | -9,19 | 3,38 |
|  |  | WT_CSD | -4,186 | 2,148 | ,393 | -10,47 | 2,10 |
|  |  | RQ_SHAM | -2,983 | 2,148 | 1,000 | -9,27 | 3,31 |
| Irg1 | WT_SHAM | WT_CSD | 1,771^*^ | ,363 | ,001 | ,71 | 2,83 |
|  |  | RQ_SHAM | ,954 | ,363 | ,097 | -,11 | 2,02 |
|  |  | RQ_CSD | -,638 | ,363 | ,567 | -1,70 | ,43 |
|  | WT_CSD | WT_SHAM | -1,771^*^ | ,363 | ,001 | -2,83 | -,71 |
|  |  | RQ_SHAM | -,818 | ,363 | ,215 | -1,88 | ,25 |
|  |  | RQ_CSD | -2,409^*^ | ,363 | ,000 | -3,47 | -1,35 |
|  | RQ_SHAM | WT_SHAM | -,954 | ,363 | ,097 | -2,02 | ,11 |
|  |  | WT_CSD | ,818 | ,363 | ,215 | -,25 | 1,88 |
|  |  | RQ_CSD | -1,591^*^ | ,363 | ,002 | -2,65 | -,53 |
|  | RQ_CSD | WT_SHAM | ,638 | ,363 | ,567 | -,43 | 1,70 |
|  |  | WT_CSD | 2,409^*^ | ,363 | ,000 | 1,35 | 3,47 |
|  |  | RQ_SHAM | 1,591^*^ | ,363 | ,002 | ,53 | 2,65 |
| Il4 | WT_SHAM | WT_CSD | -1,247 | ,569 | ,242 | -2,91 | ,42 |
|  |  | RQ_SHAM | -2,943^*^ | ,569 | ,000 | -4,61 | -1,28 |
|  |  | RQ_CSD | ,500 | ,569 | 1,000 | -1,16 | 2,17 |
|  | WT_CSD | WT_SHAM | 1,247 | ,569 | ,242 | -,42 | 2,91 |
|  |  | RQ_SHAM | -1,696^*^ | ,569 | ,044 | -3,36 | -,03 |
|  |  | RQ_CSD | 1,748^*^ | ,569 | ,036 | ,08 | 3,41 |
|  | RQ_SHAM | WT_SHAM | 2,943^*^ | ,569 | ,000 | 1,28 | 4,61 |
|  |  | WT_CSD | 1,696^*^ | ,569 | ,044 | ,03 | 3,36 |
|  |  | RQ_CSD | 3,444^*^ | ,569 | ,000 | 1,78 | 5,11 |
|  | RQ_CSD | WT_SHAM | -,500 | ,569 | 1,000 | -2,17 | 1,16 |
|  |  | WT_CSD | -1,748^*^ | ,569 | ,036 | -3,41 | -,08 |
|  |  | RQ_SHAM | -3,444^*^ | ,569 | ,000 | -5,11 | -1,78 |
| Tlr2 | WT_SHAM | WT_CSD | -9,413 | 8,637 | 1,000 | -34,69 | 15,87 |
|  |  | RQ_SHAM | 4,833 | 8,637 | 1,000 | -20,45 | 30,11 |
|  |  | RQ_CSD | -31,649^*^ | 8,637 | ,009 | -56,93 | -6,37 |
|  | WT_CSD | WT_SHAM | 9,413 | 8,637 | 1,000 | -15,87 | 34,69 |
|  |  | RQ_SHAM | 14,246 | 8,637 | ,688 | -11,04 | 39,53 |
|  |  | RQ_CSD | -22,236 | 8,637 | ,109 | -47,52 | 3,04 |
|  | RQ_SHAM | WT_SHAM | -4,833 | 8,637 | 1,000 | -30,11 | 20,45 |
|  |  | WT_CSD | -14,246 | 8,637 | ,688 | -39,53 | 11,04 |
|  |  | RQ_CSD | -36,482^*^ | 8,637 | ,002 | -61,76 | -11,20 |
|  | RQ_CSD | WT_SHAM | 31,649^*^ | 8,637 | ,009 | 6,37 | 56,93 |
|  |  | WT_CSD | 22,236 | 8,637 | ,109 | -3,04 | 47,52 |
|  |  | RQ_SHAM | 36,482^*^ | 8,637 | ,002 | 11,20 | 61,76 |
| Tlr4 | WT_SHAM | WT_CSD | -,365 | 2,353 | 1,000 | -7,25 | 6,52 |
|  |  | RQ_SHAM | -6,409 | 2,353 | ,078 | -13,30 | ,48 |
|  |  | RQ_CSD | 1,677 | 2,353 | 1,000 | -5,21 | 8,56 |
|  | WT_CSD | WT_SHAM | ,365 | 2,353 | 1,000 | -6,52 | 7,25 |
|  |  | RQ_SHAM | -6,044 | 2,353 | ,110 | -12,93 | ,84 |
|  |  | RQ_CSD | 2,042 | 2,353 | 1,000 | -4,84 | 8,93 |
|  | RQ_SHAM | WT_SHAM | 6,409 | 2,353 | ,078 | -,48 | 13,30 |
|  |  | WT_CSD | 6,044 | 2,353 | ,110 | -,84 | 12,93 |
|  |  | RQ_CSD | 8,086^*^ | 2,353 | ,016 | 1,20 | 14,97 |
|  | RQ_CSD | WT_SHAM | -1,677 | 2,353 | 1,000 | -8,56 | 5,21 |
|  |  | WT_CSD | -2,042 | 2,353 | 1,000 | -8,93 | 4,84 |
|  |  | RQ_SHAM | -8,086^*^ | 2,353 | ,016 | -14,97 | -1,20 |
| Ptgs2 | WT_SHAM | WT_CSD | 434,582^*^ | 110,665 | ,005 | 110,65 | 758,51 |
|  |  | RQ_SHAM | 339,428^*^ | 110,665 | ,036 | 15,50 | 663,36 |
|  |  | RQ_CSD | 256,847 | 110,665 | ,186 | -67,08 | 580,78 |
|  | WT_CSD | WT_SHAM | -434,582^*^ | 110,665 | ,005 | -758,51 | -110,65 |
|  |  | RQ_SHAM | -95,154 | 110,665 | 1,000 | -419,08 | 228,78 |
|  |  | RQ_CSD | -177,734 | 110,665 | ,744 | -501,66 | 146,20 |
|  | RQ_SHAM | WT_SHAM | -339,428^*^ | 110,665 | ,036 | -663,36 | -15,50 |
|  |  | WT_CSD | 95,154 | 110,665 | 1,000 | -228,78 | 419,08 |
|  |  | RQ_CSD | -82,580 | 110,665 | 1,000 | -406,51 | 241,35 |
|  | RQ_CSD | WT_SHAM | -256,847 | 110,665 | ,186 | -580,78 | 67,08 |
|  |  | WT_CSD | 177,734 | 110,665 | ,744 | -146,20 | 501,66 |
|  |  | RQ_SHAM | 82,580 | 110,665 | 1,000 | -241,35 | 406,51 |
| Il17a | WT_SHAM | WT_CSD | ,000 | ,018 | 1,000 | -,05 | ,05 |
|  |  | RQ_SHAM | ,000 | ,018 | 1,000 | -,05 | ,05 |
|  |  | RQ_CSD | -,148^*^ | ,018 | ,000 | -,20 | -,09 |
|  | WT_CSD | WT_SHAM | ,000 | ,018 | 1,000 | -,05 | ,05 |
|  |  | RQ_SHAM | ,000 | ,018 | 1,000 | -,05 | ,05 |
|  |  | RQ_CSD | -,148^*^ | ,018 | ,000 | -,20 | -,09 |
|  | RQ_SHAM | WT_SHAM | ,000 | ,018 | 1,000 | -,05 | ,05 |
|  |  | WT_CSD | ,000 | ,018 | 1,000 | -,05 | ,05 |
|  |  | RQ_CSD | -,148^*^ | ,018 | ,000 | -,20 | -,09 |
|  | RQ_CSD | WT_SHAM | ,148^*^ | ,018 | ,000 | ,09 | ,20 |
|  |  | WT_CSD | ,148^*^ | ,018 | ,000 | ,09 | ,20 |
|  |  | RQ_SHAM | ,148^*^ | ,018 | ,000 | ,09 | ,20 |
| Casp1 | WT_SHAM | WT_CSD | -6,852 | 9,690 | 1,000 | -35,21 | 21,51 |
|  |  | RQ_SHAM | -22,407 | 9,690 | ,189 | -50,77 | 5,96 |
|  |  | RQ_CSD | -15,057 | 9,690 | ,815 | -43,42 | 13,31 |
|  | WT_CSD | WT_SHAM | 6,852 | 9,690 | 1,000 | -21,51 | 35,21 |
|  |  | RQ_SHAM | -15,554 | 9,690 | ,745 | -43,92 | 12,81 |
|  |  | RQ_CSD | -8,204 | 9,690 | 1,000 | -36,57 | 20,16 |
|  | RQ_SHAM | WT_SHAM | 22,407 | 9,690 | ,189 | -5,96 | 50,77 |
|  |  | WT_CSD | 15,554 | 9,690 | ,745 | -12,81 | 43,92 |
|  |  | RQ_CSD | 7,350 | 9,690 | 1,000 | -21,01 | 35,71 |
|  | RQ_CSD | WT_SHAM | 15,057 | 9,690 | ,815 | -13,31 | 43,42 |
|  |  | WT_CSD | 8,204 | 9,690 | 1,000 | -20,16 | 36,57 |
|  |  | RQ_SHAM | -7,350 | 9,690 | 1,000 | -35,71 | 21,01 |
| Csf3 | WT_SHAM | WT_CSD | ,540 | ,359 | ,890 | -,51 | 1,59 |
|  |  | RQ_SHAM | 1,640^*^ | ,359 | ,001 | ,59 | 2,69 |
|  |  | RQ_CSD | -,121 | ,359 | 1,000 | -1,17 | ,93 |
|  | WT_CSD | WT_SHAM | -,540 | ,359 | ,890 | -1,59 | ,51 |
|  |  | RQ_SHAM | 1,100^*^ | ,359 | ,037 | ,05 | 2,15 |
|  |  | RQ_CSD | -,661 | ,359 | ,483 | -1,71 | ,39 |
|  | RQ_SHAM | WT_SHAM | -1,640^*^ | ,359 | ,001 | -2,69 | -,59 |
|  |  | WT_CSD | -1,100^*^ | ,359 | ,037 | -2,15 | -,05 |
|  |  | RQ_CSD | -1,761^*^ | ,359 | ,001 | -2,81 | -,71 |
|  | RQ_CSD | WT_SHAM | ,121 | ,359 | 1,000 | -,93 | 1,17 |
|  |  | WT_CSD | ,661 | ,359 | ,483 | -,39 | 1,71 |
|  |  | RQ_SHAM | 1,761^*^ | ,359 | ,001 | ,71 | 2,81 |
| Cxcl3 | WT_SHAM | WT_CSD | 1,363 | ,489 | ,068 | -,07 | 2,79 |
|  |  | RQ_SHAM | -,961 | ,489 | ,379 | -2,39 | ,47 |
|  |  | RQ_CSD | -1,408 | ,489 | ,055 | -2,84 | ,02 |
|  | WT_CSD | WT_SHAM | -1,363 | ,489 | ,068 | -2,79 | ,07 |
|  |  | RQ_SHAM | -2,325^*^ | ,489 | ,001 | -3,76 | -,89 |
|  |  | RQ_CSD | -2,771^*^ | ,489 | ,000 | -4,20 | -1,34 |
|  | RQ_SHAM | WT_SHAM | ,961 | ,489 | ,379 | -,47 | 2,39 |
|  |  | WT_CSD | 2,325^*^ | ,489 | ,001 | ,89 | 3,76 |
|  |  | RQ_CSD | -,446 | ,489 | 1,000 | -1,88 | ,98 |
|  | RQ_CSD | WT_SHAM | 1,408 | ,489 | ,055 | -,02 | 2,84 |
|  |  | WT_CSD | 2,771^*^ | ,489 | ,000 | 1,34 | 4,20 |
|  |  | RQ_SHAM | ,446 | ,489 | 1,000 | -,98 | 1,88 |
| Mmp9 | WT_SHAM | WT_CSD | ,020 | 3,138 | 1,000 | -9,17 | 9,21 |
|  |  | RQ_SHAM | -4,715 | 3,138 | ,892 | -13,90 | 4,47 |
|  |  | RQ_CSD | ,277 | 3,138 | 1,000 | -8,91 | 9,46 |
|  | WT_CSD | WT_SHAM | -,020 | 3,138 | 1,000 | -9,21 | 9,17 |
|  |  | RQ_SHAM | -4,735 | 3,138 | ,882 | -13,92 | 4,45 |
|  |  | RQ_CSD | ,257 | 3,138 | 1,000 | -8,93 | 9,44 |
|  | RQ_SHAM | WT_SHAM | 4,715 | 3,138 | ,892 | -4,47 | 13,90 |
|  |  | WT_CSD | 4,735 | 3,138 | ,882 | -4,45 | 13,92 |
|  |  | RQ_CSD | 4,992 | 3,138 | ,764 | -4,19 | 14,18 |
|  | RQ_CSD | WT_SHAM | -,277 | 3,138 | 1,000 | -9,46 | 8,91 |
|  |  | WT_CSD | -,257 | 3,138 | 1,000 | -9,44 | 8,93 |
|  |  | RQ_SHAM | -4,992 | 3,138 | ,764 | -14,18 | 4,19 |
| Ecm1 | WT_SHAM | WT_CSD | -19,257 | 35,168 | 1,000 | -122,20 | 83,68 |
|  |  | RQ_SHAM | -48,037 | 35,168 | 1,000 | -150,98 | 54,90 |
|  |  | RQ_CSD | -49,005 | 35,168 | 1,000 | -151,95 | 53,94 |
|  | WT_CSD | WT_SHAM | 19,257 | 35,168 | 1,000 | -83,68 | 122,20 |
|  |  | RQ_SHAM | -28,780 | 35,168 | 1,000 | -131,72 | 74,16 |
|  |  | RQ_CSD | -29,748 | 35,168 | 1,000 | -132,69 | 73,19 |
|  | RQ_SHAM | WT_SHAM | 48,037 | 35,168 | 1,000 | -54,90 | 150,98 |
|  |  | WT_CSD | 28,780 | 35,168 | 1,000 | -74,16 | 131,72 |
|  |  | RQ_CSD | -,968 | 35,168 | 1,000 | -103,91 | 101,97 |
|  | RQ_CSD | WT_SHAM | 49,005 | 35,168 | 1,000 | -53,94 | 151,95 |
|  |  | WT_CSD | 29,748 | 35,168 | 1,000 | -73,19 | 132,69 |
|  |  | RQ_SHAM | ,968 | 35,168 | 1,000 | -101,97 | 103,91 |
| Mefv | WT_SHAM | WT_CSD | ,030 | ,119 | 1,000 | -,32 | ,38 |
|  |  | RQ_SHAM | ,259 | ,119 | ,248 | -,09 | ,61 |
|  |  | RQ_CSD | -,643^*^ | ,119 | ,000 | -,99 | -,29 |
|  | WT_CSD | WT_SHAM | -,030 | ,119 | 1,000 | -,38 | ,32 |
|  |  | RQ_SHAM | ,229 | ,119 | ,409 | -,12 | ,58 |
|  |  | RQ_CSD | -,673^*^ | ,119 | ,000 | -1,02 | -,32 |
|  | RQ_SHAM | WT_SHAM | -,259 | ,119 | ,248 | -,61 | ,09 |
|  |  | WT_CSD | -,229 | ,119 | ,409 | -,58 | ,12 |
|  |  | RQ_CSD | -,902^*^ | ,119 | ,000 | -1,25 | -,55 |
|  | RQ_CSD | WT_SHAM | ,643^*^ | ,119 | ,000 | ,29 | ,99 |
|  |  | WT_CSD | ,673^*^ | ,119 | ,000 | ,32 | 1,02 |
|  |  | RQ_SHAM | ,902^*^ | ,119 | ,000 | ,55 | 1,25 |
| Csf2 | WT_SHAM | WT_CSD | ,000 | ,088 | 1,000 | -,26 | ,26 |
|  |  | RQ_SHAM | -,747^*^ | ,088 | ,000 | -1,00 | -,49 |
|  |  | RQ_CSD | ,000 | ,088 | 1,000 | -,26 | ,26 |
|  | WT_CSD | WT_SHAM | ,000 | ,088 | 1,000 | -,26 | ,26 |
|  |  | RQ_SHAM | -,747^*^ | ,088 | ,000 | -1,00 | -,49 |
|  |  | RQ_CSD | ,000 | ,088 | 1,000 | -,26 | ,26 |
|  | RQ_SHAM | WT_SHAM | ,747^*^ | ,088 | ,000 | ,49 | 1,00 |
|  |  | WT_CSD | ,747^*^ | ,088 | ,000 | ,49 | 1,00 |
|  |  | RQ_CSD | ,747^*^ | ,088 | ,000 | ,49 | 1,00 |
|  | RQ_CSD | WT_SHAM | ,000 | ,088 | 1,000 | -,26 | ,26 |
|  |  | WT_CSD | ,000 | ,088 | 1,000 | -,26 | ,26 |
|  |  | RQ_SHAM | -,747^*^ | ,088 | ,000 | -1,00 | -,49 |
| Ccr1 | WT_SHAM | WT_CSD | 1,873^*^ | ,468 | ,004 | ,50 | 3,24 |
|  |  | RQ_SHAM | 1,509^*^ | ,468 | ,025 | ,14 | 2,88 |
|  |  | RQ_CSD | ,256 | ,468 | 1,000 | -1,11 | 1,63 |
|  | WT_CSD | WT_SHAM | -1,873^*^ | ,468 | ,004 | -3,24 | -,50 |
|  |  | RQ_SHAM | -,364 | ,468 | 1,000 | -1,73 | 1,01 |
|  |  | RQ_CSD | -1,617^*^ | ,468 | ,015 | -2,99 | -,25 |
|  | RQ_SHAM | WT_SHAM | -1,509^*^ | ,468 | ,025 | -2,88 | -,14 |
|  |  | WT_CSD | ,364 | ,468 | 1,000 | -1,01 | 1,73 |
|  |  | RQ_CSD | -1,253 | ,468 | ,086 | -2,62 | ,12 |
|  | RQ_CSD | WT_SHAM | -,256 | ,468 | 1,000 | -1,63 | 1,11 |
|  |  | WT_CSD | 1,617^*^ | ,468 | ,015 | ,25 | 2,99 |
|  |  | RQ_SHAM | 1,253 | ,468 | ,086 | -,12 | 2,62 |
| Socs3 | WT_SHAM | WT_CSD | 167,315 | 89,602 | ,460 | -94,96 | 429,59 |
|  |  | RQ_SHAM | 159,729 | 89,602 | ,539 | -102,55 | 422,00 |
|  |  | RQ_CSD | -176,550 | 89,602 | ,377 | -438,83 | 85,73 |
|  | WT_CSD | WT_SHAM | -167,315 | 89,602 | ,460 | -429,59 | 94,96 |
|  |  | RQ_SHAM | -7,586 | 89,602 | 1,000 | -269,86 | 254,69 |
|  |  | RQ_CSD | -343,865^*^ | 89,602 | ,006 | -606,14 | -81,59 |
|  | RQ_SHAM | WT_SHAM | -159,729 | 89,602 | ,539 | -422,00 | 102,55 |
|  |  | WT_CSD | 7,586 | 89,602 | 1,000 | -254,69 | 269,86 |
|  |  | RQ_CSD | -336,279^*^ | 89,602 | ,008 | -598,55 | -74,00 |
|  | RQ_CSD | WT_SHAM | 176,550 | 89,602 | ,377 | -85,73 | 438,83 |
|  |  | WT_CSD | 343,865^*^ | 89,602 | ,006 | 81,59 | 606,14 |
|  |  | RQ_SHAM | 336,279^*^ | 89,602 | ,008 | 74,00 | 598,55 |
| Saa3 | WT_SHAM | WT_CSD | -1,560^*^ | ,496 | ,031 | -3,01 | -,11 |
|  |  | RQ_SHAM | ,000 | ,496 | 1,000 | -1,45 | 1,45 |
|  |  | RQ_CSD | -3,620^*^ | ,496 | ,000 | -5,07 | -2,17 |
|  | WT_CSD | WT_SHAM | 1,560^*^ | ,496 | ,031 | ,11 | 3,01 |
|  |  | RQ_SHAM | 1,560^*^ | ,496 | ,031 | ,11 | 3,01 |
|  |  | RQ_CSD | -2,059^*^ | ,496 | ,003 | -3,51 | -,61 |
|  | RQ_SHAM | WT_SHAM | ,000 | ,496 | 1,000 | -1,45 | 1,45 |
|  |  | WT_CSD | -1,560^*^ | ,496 | ,031 | -3,01 | -,11 |
|  |  | RQ_CSD | -3,620^*^ | ,496 | ,000 | -5,07 | -2,17 |
|  | RQ_CSD | WT_SHAM | 3,620^*^ | ,496 | ,000 | 2,17 | 5,07 |
|  |  | WT_CSD | 2,059^*^ | ,496 | ,003 | ,61 | 3,51 |
|  |  | RQ_SHAM | 3,620^*^ | ,496 | ,000 | 2,17 | 5,07 |
| Tnfaip6 | WT_SHAM | WT_CSD | 218,543 | 76,997 | ,061 | -6,84 | 443,92 |
|  |  | RQ_SHAM | 200,350 | 76,997 | ,102 | -25,03 | 425,73 |
|  |  | RQ_CSD | 179,756 | 76,997 | ,181 | -45,62 | 405,13 |
|  | WT_CSD | WT_SHAM | -218,543 | 76,997 | ,061 | -443,92 | 6,84 |
|  |  | RQ_SHAM | -18,193 | 76,997 | 1,000 | -243,57 | 207,19 |
|  |  | RQ_CSD | -38,787 | 76,997 | 1,000 | -264,17 | 186,59 |
|  | RQ_SHAM | WT_SHAM | -200,350 | 76,997 | ,102 | -425,73 | 25,03 |
|  |  | WT_CSD | 18,193 | 76,997 | 1,000 | -207,19 | 243,57 |
|  |  | RQ_CSD | -20,594 | 76,997 | 1,000 | -245,97 | 204,79 |
|  | RQ_CSD | WT_SHAM | -179,756 | 76,997 | ,181 | -405,13 | 45,62 |
|  |  | WT_CSD | 38,787 | 76,997 | 1,000 | -186,59 | 264,17 |
|  |  | RQ_SHAM | 20,594 | 76,997 | 1,000 | -204,79 | 245,97 |
| Il13 | WT_SHAM | WT_CSD | -1,036^*^ | ,175 | ,000 | -1,55 | -,52 |
|  |  | RQ_SHAM | -,018 | ,175 | 1,000 | -,53 | ,50 |
|  |  | RQ_CSD | -,289 | ,175 | ,692 | -,80 | ,22 |
|  | WT_CSD | WT_SHAM | 1,036^*^ | ,175 | ,000 | ,52 | 1,55 |
|  |  | RQ_SHAM | 1,018^*^ | ,175 | ,000 | ,50 | 1,53 |
|  |  | RQ_CSD | ,747^*^ | ,175 | ,002 | ,23 | 1,26 |
|  | RQ_SHAM | WT_SHAM | ,018 | ,175 | 1,000 | -,50 | ,53 |
|  |  | WT_CSD | -1,018^*^ | ,175 | ,000 | -1,53 | -,50 |
|  |  | RQ_CSD | -,271 | ,175 | ,828 | -,78 | ,24 |
|  | RQ_CSD | WT_SHAM | ,289 | ,175 | ,692 | -,22 | ,80 |
|  |  | WT_CSD | -,747^*^ | ,175 | ,002 | -1,26 | -,23 |
|  |  | RQ_SHAM | ,271 | ,175 | ,828 | -,24 | ,78 |
| Clec4e | WT_SHAM | WT_CSD | 3,780^*^ | ,659 | ,000 | 1,85 | 5,71 |
|  |  | RQ_SHAM | 2,625^*^ | ,659 | ,004 | ,70 | 4,56 |
|  |  | RQ_CSD | ,518 | ,659 | 1,000 | -1,41 | 2,45 |
|  | WT_CSD | WT_SHAM | -3,780^*^ | ,659 | ,000 | -5,71 | -1,85 |
|  |  | RQ_SHAM | -1,155 | ,659 | ,571 | -3,08 | ,77 |
|  |  | RQ_CSD | -3,262^*^ | ,659 | ,000 | -5,19 | -1,33 |
|  | RQ_SHAM | WT_SHAM | -2,625^*^ | ,659 | ,004 | -4,56 | -,70 |
|  |  | WT_CSD | 1,155 | ,659 | ,571 | -,77 | 3,08 |
|  |  | RQ_CSD | -2,107^*^ | ,659 | ,027 | -4,04 | -,18 |
|  | RQ_CSD | WT_SHAM | -,518 | ,659 | 1,000 | -2,45 | 1,41 |
|  |  | WT_CSD | 3,262^*^ | ,659 | ,000 | 1,33 | 5,19 |
|  |  | RQ_SHAM | 2,107^*^ | ,659 | ,027 | ,18 | 4,04 |
| Il2 | WT_SHAM | WT_CSD | -1,196^*^ | ,164 | ,000 | -1,68 | -,72 |
|  |  | RQ_SHAM | ,000 | ,164 | 1,000 | -,48 | ,48 |
|  |  | RQ_CSD | ,000 | ,164 | 1,000 | -,48 | ,48 |
|  | WT_CSD | WT_SHAM | 1,196^*^ | ,164 | ,000 | ,72 | 1,68 |
|  |  | RQ_SHAM | 1,196^*^ | ,164 | ,000 | ,72 | 1,68 |
|  |  | RQ_CSD | 1,196^*^ | ,164 | ,000 | ,72 | 1,68 |
|  | RQ_SHAM | WT_SHAM | ,000 | ,164 | 1,000 | -,48 | ,48 |
|  |  | WT_CSD | -1,196^*^ | ,164 | ,000 | -1,68 | -,72 |
|  |  | RQ_CSD | ,000 | ,164 | 1,000 | -,48 | ,48 |
|  | RQ_CSD | WT_SHAM | ,000 | ,164 | 1,000 | -,48 | ,48 |
|  |  | WT_CSD | -1,196^*^ | ,164 | ,000 | -1,68 | -,72 |
|  |  | RQ_SHAM | ,000 | ,164 | 1,000 | -,48 | ,48 |
| Tnfaip3 | WT_SHAM | WT_CSD | -3,571 | 10,119 | 1,000 | -33,19 | 26,05 |
|  |  | RQ_SHAM | -8,127 | 10,119 | 1,000 | -37,75 | 21,49 |
|  |  | RQ_CSD | -7,292 | 10,119 | 1,000 | -36,91 | 22,33 |
|  | WT_CSD | WT_SHAM | 3,571 | 10,119 | 1,000 | -26,05 | 33,19 |
|  |  | RQ_SHAM | -4,556 | 10,119 | 1,000 | -34,18 | 25,06 |
|  |  | RQ_CSD | -3,721 | 10,119 | 1,000 | -33,34 | 25,90 |
|  | RQ_SHAM | WT_SHAM | 8,127 | 10,119 | 1,000 | -21,49 | 37,75 |
|  |  | WT_CSD | 4,556 | 10,119 | 1,000 | -25,06 | 34,18 |
|  |  | RQ_CSD | ,835 | 10,119 | 1,000 | -28,78 | 30,45 |
|  | RQ_CSD | WT_SHAM | 7,292 | 10,119 | 1,000 | -22,33 | 36,91 |
|  |  | WT_CSD | 3,721 | 10,119 | 1,000 | -25,90 | 33,34 |
|  |  | RQ_SHAM | -,835 | 10,119 | 1,000 | -30,45 | 28,78 |
| Myd88 | WT_SHAM | WT_CSD | 43,575 | 61,129 | 1,000 | -135,36 | 222,51 |
|  |  | RQ_SHAM | 41,201 | 61,129 | 1,000 | -137,73 | 220,13 |
|  |  | RQ_CSD | -58,330 | 61,129 | 1,000 | -237,26 | 120,60 |
|  | WT_CSD | WT_SHAM | -43,575 | 61,129 | 1,000 | -222,51 | 135,36 |
|  |  | RQ_SHAM | -2,374 | 61,129 | 1,000 | -181,31 | 176,56 |
|  |  | RQ_CSD | -101,905 | 61,129 | ,667 | -280,84 | 77,03 |
|  | RQ_SHAM | WT_SHAM | -41,201 | 61,129 | 1,000 | -220,13 | 137,73 |
|  |  | WT_CSD | 2,374 | 61,129 | 1,000 | -176,56 | 181,31 |
|  |  | RQ_CSD | -99,531 | 61,129 | ,715 | -278,46 | 79,40 |
|  | RQ_CSD | WT_SHAM | 58,330 | 61,129 | 1,000 | -120,60 | 237,26 |
|  |  | WT_CSD | 101,905 | 61,129 | ,667 | -77,03 | 280,84 |
|  |  | RQ_SHAM | 99,531 | 61,129 | ,715 | -79,40 | 278,46 |
| Cxcl9 | WT_SHAM | WT_CSD | ,441 | ,504 | 1,000 | -1,04 | 1,92 |
|  |  | RQ_SHAM | ,398 | ,504 | 1,000 | -1,08 | 1,87 |
|  |  | RQ_CSD | -3,222^*^ | ,504 | ,000 | -4,70 | -1,75 |
|  | WT_CSD | WT_SHAM | -,441 | ,504 | 1,000 | -1,92 | 1,04 |
|  |  | RQ_SHAM | -,043 | ,504 | 1,000 | -1,52 | 1,43 |
|  |  | RQ_CSD | -3,662^*^ | ,504 | ,000 | -5,14 | -2,19 |
|  | RQ_SHAM | WT_SHAM | -,398 | ,504 | 1,000 | -1,87 | 1,08 |
|  |  | WT_CSD | ,043 | ,504 | 1,000 | -1,43 | 1,52 |
|  |  | RQ_CSD | -3,620^*^ | ,504 | ,000 | -5,10 | -2,14 |
|  | RQ_CSD | WT_SHAM | 3,222^*^ | ,504 | ,000 | 1,75 | 4,70 |
|  |  | WT_CSD | 3,662^*^ | ,504 | ,000 | 2,19 | 5,14 |
|  |  | RQ_SHAM | 3,620^*^ | ,504 | ,000 | 2,14 | 5,10 |

| *. La differenza della media è significativa al livello 0.05. |
| --- |
